# Bridging the Gap Between Awareness and Behaviour in Pressure Injury Prevention: A Cross‐Sectional Survey of Self‐Management in People With Spinal Cord Injury

**DOI:** 10.1111/iwj.71049

**Published:** 2026-09-18

**Authors:** Liang Qin Liu, Evangeline Martinez, Jacinta Kelly, Helen Allan, Sarah Knight

**Affiliations:** ^1^ Department of Nursing and Midwifery, Faculty of Health, Social Care and Education Middlesex University London UK; ^2^ London Spinal Cord Injury Centre Royal National Orthopaedic Hospital Stanmore UK

**Keywords:** illness perception (mBIPQ), pressure injury, pressure‐relief exercise, self‐management, spinal cord injury

## Abstract

To examine how self‐management attitudes, illness perceptions and preventive behaviours relate to adherence to pressure injury prevention in people with spinal cord injury (PwSCI) using a theory‐informed framework. A cross‐sectional survey of 184 wheelchair‐users with a SCI within community and specialist SCI centres. Preventive behaviours (pressure relief, skin inspection), self‐management domains (concordance, perceived benefits, perceived negatives, practical barriers) and illness perceptions (mBIPQ) were assessed. Associations and subgroup differences by pressure injury history and education were analysed. Participants reported high perceived benefit of pressure‐relief activities (4.02 ± 0.62) but only moderate concordance (3.05 ± 0.79) and persistent practical barriers (3.22 ± 0.75), indicating a gap between awareness and routine behaviour. Concordance was positively associated with pressure‐relief activity (*r* = 0.49, *p* < 0.001) and negatively with barriers (*r* = −0.56, *p* < 0.001). Prior pressure injury education and experience were associated with more positive attitudes and greater engagement. Older age (≥ 55 years), but not injury duration, was associated with higher adherence. Despite high awareness, adherence to preventive routines remains constrained by perceptual and practical barriers. This study provides quantitative evidence explaining why awareness does not consistently translate into preventive behaviour, highlighting the need to address real‐world barriers beyond education alone.

## Key Points

- High awareness of pressure injury risk did not consistently translate into preventive behaviour.
- Greater concordance was associated with more frequent pressure‐relief activity, while practical barriers and negative perceptions were associated with lower engagement.
- Previous pressure injury education and lived experience of pressure injury were associated with more positive preventive attitudes and behaviours.
- Pressure injury prevention should extend beyond education alone to address individual beliefs, practical barriers and everyday care circumstances.

**Abbreviations**
ANOVA: analysis of variance
ASPIRE: Association for Spinal Injury Research, Rehabilitation and Reintegration
CI: confidence interval
CPMS: central portfolio management system
CSM: common‐sense model
HBM: health belief model
HRA: health research authority
IRAS: integrated research application system
KMO: Kaiser–Meyer–Olkin measure of sampling adequacy
mBIPQ: modified brief illness perception questionnaire
NHS: National Health Service
NIHR: National Institute for Health and Care Research
NSCISC: National Spinal Cord Injury Statistical Center
PPA: perceptions and practicalities approach
PwSCI: people with spinal cord injury
REC: research ethics committee
SCI: spinal cord injury
SIA: spinal injuries association
SPSS: statistical package for the social sciences
TDF: theoretical domains framework

## 1 Introduction

Globally, more than 15 million people live with a spinal cord injury (PwSCI) [1]. In the UK, approximately 110 000 individuals live with SCI, with around 4400 new cases annually [2]. Pressure injuries remain one of the most frequent and serious secondary complications, affecting 20%–30% of PwSCI within 1–5 years post‐injury and up to 85% over a lifetime [2, 3, 4, 5].

Pressure injury, also referred to as pressure ulcer, bedsores or decubitus ulcers, is defined as localised tissue damage resulting from sustained pressure and shear. The European Pressure Ulcer Advisory Panel [6] categorises pressure injury into four stages ranging from non‐blanchable erythema to full‐thickness tissue loss. Following SCI, loss of motor and sensory function, reduced mobility and muscle atrophy increase vulnerability to pressure‐related tissue injury, particularly over the sacrum and ischial tuberosities [7].

Pressure injuries represent a substantial health, social and economic burden for PwSCI. Pressure injuries are the second leading cause of rehospitalisation in this population [3]. Recurrence rates are substantial and highly variable (5.4%–73.6%), with individuals with SCI consistently identified as a high‐risk group [8]. They are also consistently associated with an increased risk of mortality in PwSCI [9]. Once a pressure injury is established, it is extremely difficult to fully heal due to impaired perfusion, recurrent pressure and compromised tissue integrity. The development of a pressure injury during the acute post‐injury phase can lead to prolonged hospitalisation and delayed rehabilitation, in addition to the already significant challenges faced early after an SCI, including medical instability, impaired mobility and sensation, high vulnerability to secondary complications and substantial psychological and caregiving demands. In the longer term, severe or recurrent pressure injuries can cause prolonged periods of bedrest, reduce quality of life, require complex surgical interventions, and, in some extreme cases, result in life‐threatening sepsis [3, 10, 11].

Economically, pressure injuries pose a significant challenge to healthcare systems. It is estimated that they account for 25% of healthcare expenditures related to SCI. The cost of treating a single Grade IV pressure injury in the UK averages £14 108. The total annual cost of pressure injury treatment ranges from £1.4 to £2.1 billion, representing approximately 4% of the NHS budget [8, 12, 13].

Given the significant detrimental consequences and financial burden, prevention of a pressure injury is vitally important in the SCI population. Education of patients to increase their knowledge of skin care and self‐management regimen remains a fundamental component to reduce pressure injury incidence. Following an SCI, among many other training programmes for patients gaining skills and knowledge of SCI to adapt post‐injury daily living, patients are provided with education and training in hospital during the rehabilitation stage regarding skincare for prevention of pressure injury. For example, patients are advised to adopt a healthy lifestyle, inspect skin over bony areas and perform pressure‐relief regularly. Yet previous studies indicate that individuals with SCI often perform skincare regimens inconsistently after discharge to the community [11, 14, 15, 16, 17]. Pressure injury incidence remains high among PwSCI. The reasons why some individuals adhere to skincare self‐management routines while others do not remain poorly understood.

The Health Belief Model (HBM) proposes that preventive behaviour is shaped by perceived susceptibility, severity, benefits and barriers [18, 19], while the Perceptions and Practicalities Approach (PPA) emphasises the interaction between perceptual factors (beliefs about necessity and concerns) and practical constraints (e.g., physical limitations, time, environmental barriers) [20, 21]. Although these frameworks are widely applied in chronic disease self‐management, their combined application to pressure injury prevention in SCI has been limited, particularly in quantitative research.

Existing work in SCI has largely relied on qualitative approaches to describe lived experiences of prevention, highlighting the importance of motivation, environmental constraints and emotional responses to risk [22, 23]. However, there is limited quantitative evidence linking these perceptual and practical factors to measurable preventive behaviours. As a result, the mechanisms underlying the gap between awareness and adherence remain poorly characterised.

This study addresses this gap by integrating behavioural theory with quantitative analysis to identify mechanisms underlying the gap between awareness and adherence in pressure injury prevention.

Specifically, we aimed to: (1) quantify self‐management attitudes and pressure injury illness perceptions; (2) examine their associations with preventive behaviours; and (3) identify how these relationships differ according to demographic and clinical factors. By integrating both perceptual and practical factors together, this study seeks to provide useful insight into why awareness does not consistently translate into preventive action.

## 2 Methods

### 2.1 Study Design and Ethical Approval

This cross‐sectional quantitative study examined attitudes, behaviours and pressure injury illness perceptions among PwSCI. Ethical approval was granted by the Health and Social Care Ethics Sub‐Committee at a national university (Ref: 25745), and by the National Research Ethics Committee/Health Research Authority (IRAS ID: 330404; REC Ref: 23/WM/0240). The study was registered on the NIHR Clinical Research Network Portfolio (CPMS ID: 60009).

Data were collected between February and September 2024 using SurveyMonkey. Participants viewed an online information sheet describing the study purpose, procedures and confidentiality safeguards prior to participation. Continuing beyond the information page constituted informed consent. Participants could withdraw at any time by exiting the questionnaire.

### 2.2 Participants and Recruitment

A pragmatic recruitment approach, commonly used in health‐related cross‐sectional and exploratory behavioural research, was adopted. Eligible participants were wheelchair‐user adults with an SCI of at least 6 months' duration, regardless of neurological level. Participants were recruited through a large SCI specialist rehabilitation centre and national SCI charities (the Spinal Injuries Association [SIA] and ASPIRE) to ensure efficient access to community‐dwelling individuals with varied injury levels and durations. Research nurses (EM) screened hospital patients, while community recruitment was facilitated through charity websites, newsletters and social media. Participants completed the questionnaire online via a survey link or QR code, or requested a paper version.

Completion time was approximately 10–15 min. A pilot test with 20 PwSCI assessed clarity and feasibility of the questionnaire. No amendments were required, and pilot data were included in the final dataset.

Due to the anonymous multi‐channel recruitment strategy including charity dissemination and open survey links, it was not possible to collect data on non‐respondents or directly compare baseline characteristics between respondents and non‐respondents. This limitation is inherent in community‐based survey designs but enabled broader inclusion of individuals outside clinical registries.

### 2.3 Survey Questionnaire Measures

The questionnaire comprised four components: (i) demographic characteristics; (ii) skincare self‐management behaviours; (iii) four self‐management attitude domains; and (iv) pressure injury illness perception.

Demographic items captured age, sex, ethnicity, injury level and duration and previous pressure injury history. Behavioural items recorded the number of days participants performed skin inspection and the extent of pressure‐relief activity during the previous week.

Self‐management attitudes were informed by the HBM and Perceptions and Practicalities Approach PPA and had been preliminarily validated previously [14]. It was assessed using 26 items [14, 24] covering four domains: concordance, perceived benefits, perceived negative consequences and practical barriers. Domain scores (range 1–5) were derived from item means, with higher values indicating greater adherence (concordance), stronger perceived benefit, greater concern (negatives) or more obstacles (practical barriers).

Concordance was assessed using the six‐item domain of the previously developed SCI‐specific pressure‐relief questionnaire [14]. The items assess the frequency of reported departures from instructed wheelchair pressure‐relief activities, including performing activities for a shorter duration or less frequently than instructed, accidentally missing or deciding not to perform them, using a different type of pressure relief and stopping activities at times. Responses are recorded on a five‐point scale ranging from Always to Never, with higher scores indicating fewer reported departures from instructed activities. The questionnaire does not establish whether instructions were individually tailored or mutually agreed, nor does it assess the quality of shared decision‐making. Accordingly, concordance is operationalised in this study as reported alignment with instructed activities, rather than as a measure of an individual's motivation or willingness to engage in prevention. The complete questionnaire is provided in Appendix S1.

Pressure injury illness perception was measured using the modified Brief Illness Perception Questionnaire (mBIPQ) [25], an eight‐item instrument capturing cognitive and emotional representations of pressure injury. Total scores ranged from 0 to 80, with higher scores indicating greater perceived threat.

### 2.4 Data Analysis

As this study employed a pragmatic, cross‐sectional design with multi‐channel recruitment, no formal a priori sample size calculation was conducted. However, the achieved sample size (*N* = 184) is considered adequate for the planned analyses. For group comparisons, this sample provides sufficient power (> 80%) to detect moderate effect sizes (Cohen's d ≈ 0.4–0.5) at *α* = 0.05. For exploratory factor analysis, commonly recommended subject‐to‐item ratios (≥ 5–10 participants per item) were met, supporting the stability of the factor structure. Therefore, the sample size was deemed appropriate for both psychometric evaluation and between‐group comparisons.

Survey data were exported from SurveyMonkey, cleaned in Microsoft Excel and analysed using IBM SPSS Statistics (Version 29, Armonk, New York, USA). Descriptive statistics summarised sample characteristics and scale scores. Normality was assessed using the Kolmogorov–Smirnov test. Missing data were minimal (< 5%) across all variables and were handled using pairwise deletion to maximise available information. No systematic patterns were observed.

Depending on distribution, group comparisons used independent *t*‐tests or one‐way ANOVA for normal data and Mann–Whitney U or Kruskal–Wallis H tests for non‐normal data. Correlations between attitudes, behaviours and illness‐perception domains were examined using Pearson's or Spearman's coefficients. All tests were two‐tailed with significance set at *p* < 0.05.

## 3 Results

### 3.1 Sociodemographic Characteristics

Between February and September 2024, 208 unique individuals accessed the survey platform. Of these, 184 participants completed ≥ 90% of key items of the questionnaire and were included in the final analysis (completion rate: 88.5%). This predefined threshold ensured adequate data completeness for reliable analysis. A total of 24 responses were incomplete and excluded based on predefined data completeness criteria.

Among 184 participants included in the analysis, the mean age was 46.8 ± 13.2 years; 111 (60.3%) were male and 72 (39.2%) were female. The majority had thoracic‐level injuries (*n* = 99; 53.8%), followed by cervical (*n* = 42; 22.8%) and lumbar/sacral injuries (*n* = 40; 21.7%). The mean duration of SCI was 12.7 ± 9.4 years. More than half of participants (*n* = 107; 58.2%) reported a previous history of pressure ulcers, and *n* = 81 (44.0%) had received prior education on pressure injury prevention. Table 1 summarises the participants' demographic and clinical characteristics.

**TABLE 1:** Characteristics of all participants who completed the questionnaire (*N* = 184).

Characteristic	*n* (%) or Mean ± SD
Age (years)	46.8 ± 13.2 (range 18–65+)
18–24	16 (8.7)
25–34	35 (19.0)
35–44	46 (25.0)
45–54	44 (23.9)
55–64	31 (16.8)
≥ 65	12 (6.5)
Sex	
Male	111 (60.3)
Female	72 (39.2)
Ethnic group	
White	118 (64.1)
Black/African/Caribbean	27 (14.7)
Asian	23 (12.5)
Mixed/other	14 (7.6)
Marital status	
Single/never married	79 (43.0)
Married/partnered	67 (36.5)
Divorced/widowed	36 (19.6)
Educational qualification	
Secondary or below	46 (25.0)
College/diploma	71 (38.6)
University or above	66 (35.9)
Smoking status	
Current smoker	48 (26.1)
Former/never smoker	134 (72.8)
Duration of injury (years)	12.7 ± 9.4
Level of injury	
Cervical	42 (22.8)
Thoracic	99 (53.8)
Lumbar/sacral	40 (21.7)
Pressure injury history	
Yes	107 (58.2)
No	77 (41.8)
Prior pressure injury education	
Yes	81 (44.0)
No	103 (56.0)

### 3.2 Reliability and Validity of the Self‐Management Scales

Before exploring group differences and behavioural associations, the psychometric properties of the four self‐management domains: Concordance, Perceived Benefit, Perceived Negatives and Practical Barriers were examined. All domains demonstrated acceptable to good internal consistency (Cronbach's *α* = 0.77–0.84) and unidimensional factor structures explaining 47%–51% of the total variance. Sampling adequacy was strong (all KMO ≥ 0.76), and Bartlett's tests confirmed factorability (all *p* < 0.001). Inter‐domain correlations supported construct validity, with moderate negative associations between concordance and perceived negatives or practical barriers, and a strong positive association between perceived negatives and barriers.

These findings confirm the reliability and validity preliminary evaluation reported in previous publication [14], which established the initial reliability and conceptual framework of the self‐management scale among individuals with SCI. The present analysis, conducted in a larger and more diverse sample, provides further evidence of internal consistency, factorial validity and conceptual distinctiveness of the four attitudinal domains, supporting their suitability for use in quantitative research and clinical assessment of pressure injury prevention self‐management.

### 3.3 Self‐Management and Illness‐Perception Scores

Across the study sample, participants demonstrated generally positive attitudes towards pressure ulcer prevention, with moderate variability across domains. The mean Concordance score was 3.05 ± 0.79 (range = 1–5), indicating moderate adherence to recommended pressure‐relief behaviours. Perceived Benefit showed the highest overall rating (4.02 ± 0.62, range = 1.86–5), suggesting that most participants recognised clear advantages of engaging in preventive activities. In contrast, both Perceived Negative (3.12 ± 0.77, range = 1–5) and Practical Barrier (3.22 ± 0.75, range = 1–5) scores were moderate, reflecting some perceived challenges and negative feelings associated with regular pressure‐relief practices. The Illness Perception (MBIPQ) score averaged 55.99 ± 10.83 (range = 7.8–77), indicating considerable variation in how participants understood and perceived their condition. Table 2 shows attitude and mIPQ scores.

**TABLE 2:** Attitude domain and pressure injury illness‐perception scores among 184 participants.

Domain	Mean ± SD	Range
Concordance	3.05 ± 0.79	1.00–5.00
Perceived benefit	4.02 ± 0.62	1.86–5.00
Perceived negatives	3.12 ± 0.77	1.00–4.67
Practical barriers	3.22 ± 0.75	1.00–5.00
Illness perception (MBIPQ)	55.99 ± 10.83	7.80–77.00

Overall, these findings suggest that while participants generally recognised the benefits of preventive activities, perceived barriers and practical challenges still influenced their level of concordance with recommended pressure ulcer prevention strategies.

### 3.4 Correlations Among Attitude Domains and Illness Perception

Concordance demonstrated moderate‐to‐strong negative correlations with both perceived negatives (*r* = −0.53, *p* < 0.001) and practical barriers (*r* = −0.56, *p* < 0.001), indicating that individuals with higher levels of concordance were less likely to endorse negative beliefs or report barriers to preventive behaviours. In contrast, perceived negatives and practical barriers were strongly positively correlated (*r* = 0.71, *p* < 0.001), suggesting that these constructs tend to co‐occur and may reinforce each other in shaping behaviour.

Perceived benefit showed a moderate positive correlation with MBIPQ scores (*r* = 0.32, *p* < 0.001), indicating that individuals who perceived greater benefits of preventive actions also reported more adaptive illness perceptions. Full correlation coefficients are presented in Appendix S2.

Overall, these findings suggest a coherent pattern in which more positive attitudes reflected by higher concordance and perceived benefit are associated with fewer negative beliefs and reduced perceived barriers. This highlights the potential role of cognitive and behavioural factors in shaping preventive self‐management.

### 3.5 Correlations Between Attitudes and Preventive Behaviours

Greater engagement in pressure‐relief exercise was positively associated with concordance (*r* = 0.49, *p* < 0.001), indicating that individuals whose attitudes were more aligned with recommended preventive behaviours were more likely to regularly perform pressure‐relief activities. In contrast, engagement was negatively associated with perceived barriers (*r* = −0.34, *p* < 0.001) and perceived negatives (*r* = −0.22, *p* = 0.004), suggesting that individuals who reported fewer practical obstacles and held fewer negative beliefs were more likely to adopt these behaviours. A small but significant positive correlation was also observed with perceived benefit (*r* = 0.18, *p* = 0.018), indicating that recognising the value of preventive actions may further support engagement.

In contrast, skin‐inspection frequency showed no significant correlation with any of the attitudinal domains (Appendix S3). This lack of association suggests that, unlike pressure‐relief exercise, skin inspection may be less influenced by attitudes and perceptions, and instead driven by other factors such as routine, caregiver support, clinical advice or habit formation.

Overall, these findings indicate that pressure‐relief exercise is more closely linked to attitudinal and perceptual factors, whereas skin inspection behaviour may be driven through different behavioural mechanisms.

### 3.6 Influence of Age, Level and Duration of Injury

**FIGURE 1:**
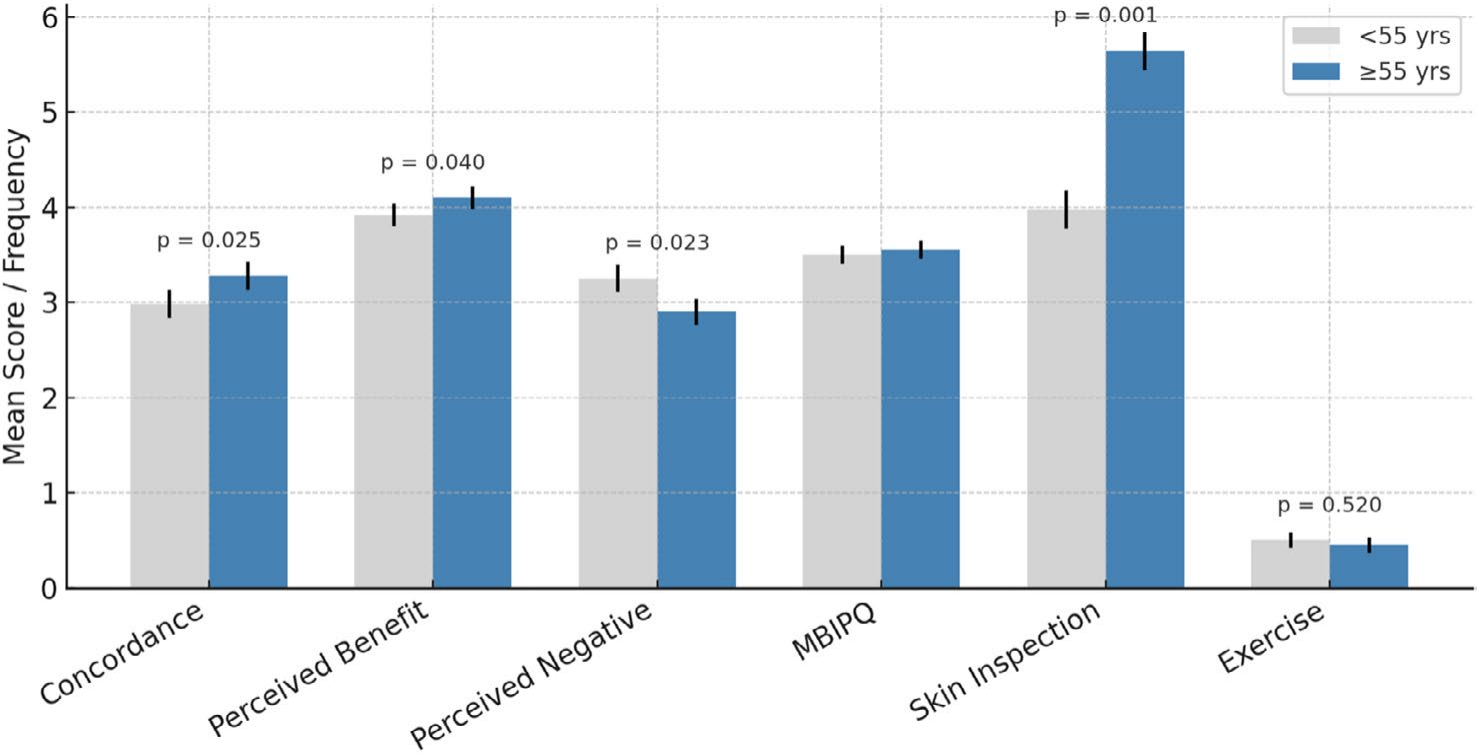
Differences in attitudes and behaviours by age group.

No significant differences across multiple age categories were observed. However, when dichotomised, adults aged ≥ 55 years demonstrated significantly more positive attitudes: higher Concordance (3.28 ± 0.69 vs. 2.98 ± 0.80; 95% CI 0.08–0.54; *p* = 0.025), higher Perceived Benefit (4.10 ± 0.57 vs. 3.92 ± 0.64; 95% CI 0.01–0.35; *p* = 0.04) and lower Perceived Negatives (2.90 ± 0.74 vs. 3.25 ± 0.77; 95% CI −0.63 to −0.06; *p* = 0.023). MBIPQ scores did not differ significantly. Adults aged ≥ 55 years also performed more frequent skin inspection (5.64 ± 2.31 vs. 3.97 ± 1.95 days/week; mean difference −1.68 days, 95% CI −2.59 to −0.76; *p* = 0.0010), whereas pressure‐relief exercise did not differ (*p* = 0.52) (Figure 1).

Injury duration was not significantly associated with concordance, perceived negatives or barriers. Comparisons across neurological levels (cervical, thoracic, lumbar/sacral) revealed no significant differences in attitudes, behaviours or illness perceptions, suggesting that age, not injury level or duration, was most consistently associated with preventive attitudes and behaviours. The lack of association between injury duration and adherence should be interpreted cautiously, as duration is inherently confounded with age.

### 3.7 Attitudes and Behaviours by Prior Pressure Injury Education

Participants with previous pressure injury education demonstrated more positive attitudes and stronger engagement. They reported higher concordance (3.18 ± 0.78 vs. 2.86 ± 0.72; 95% CI 0.05–0.59; *p* = 0.02), lower perceived negatives (2.96 ± 0.76 vs. 3.44 ± 0.73; 95% CI −0.73 to −0.23; *p* < 0.001) and lower practical barriers (3.07 ± 0.76 vs. 3.48 ± 0.69; 95% CI −0.67 to −0.16; *p* < 0.001). Illness‐perception (MBIPQ) scores were also higher (59.3 ± 10.4 vs. 53.4 ± 10.8; 95% CI 2.1–9.8; *p* = 0.003). Perceived benefit did not differ (*p* = 0.31).

**FIGURE 2:**
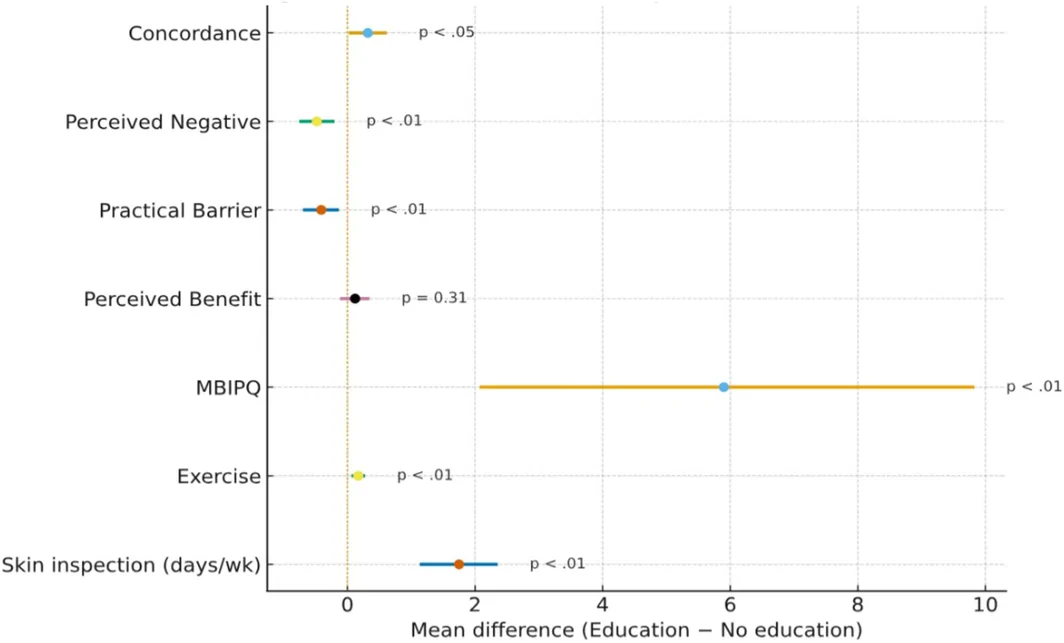
Mean differences (95% CI) in attitude and behaviour scores between participants with and without prior pressure injury education.

Behaviourally, participants with prior education performed more pressure‐relief exercise (0.58 ± 0.28 vs. 0.41 ± 0.24; mean difference 0.17, 95% CI 0.09–0.25; *p* < 0.001) and more frequent skin inspection (5.21 ± 2.04 vs. 3.46 ± 1.81 days/week; mean difference 1.74, 95% CI 1.16–2.33; *p* < 0.001). These findings underscore the strong influence of structured education on preventive engagement (Figure 2).

### 3.8 Attitudes and Behaviours by Pressure Injury History

Participants with previous pressure injury history demonstrated more positive attitudes towards prevention. They showed higher concordance (3.17 ± 0.79 vs. 2.87 ± 0.72; 95% CI 0.06–0.52; *p* = 0.011), fewer perceived negatives (2.96 ± 0.76 vs. 3.37 ± 0.75; 95% CI −0.63 to −0.17; *p* = 0.0007) and fewer practical barriers (3.07 ± 0.76 vs. 3.44 ± 0.69; 95% CI −0.59 to −0.15; *p* = 0.0017).

**FIGURE 3:**
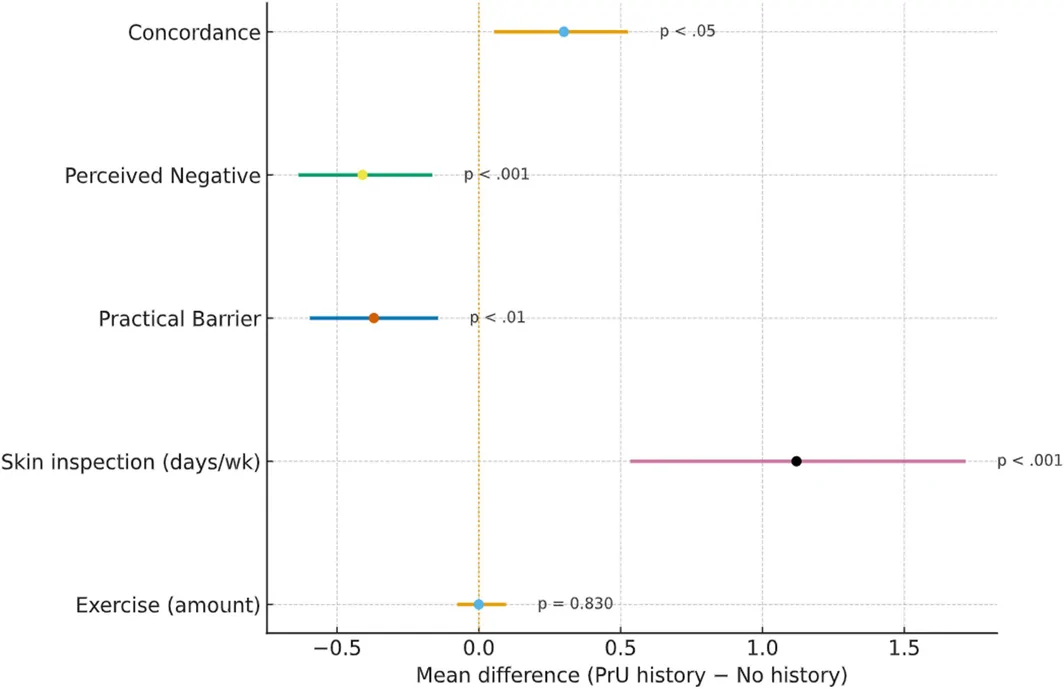
Mean differences (95% CI) in attitude and behaviour scores between participants with and without a history of pressure injury.

They also performed skin inspection more frequently (4.64 ± 2.20 vs. 3.52 ± 1.69 days/week; mean difference 1.12 days, 95% CI 0.54–1.71; *p* < 0.001). Pressure‐relief exercise did not differ (*p* = 0.83) (Figure 3).

Overall, PwSCI with lived pressure injury experience demonstrated greater vigilance and more favourable preventive attitudes, indicating that previous ulcer development may act as a strong cue to action.

### 3.9 Educational Qualification and Ethnic Group Comparisons

Educational qualification was associated with concordance (*p* = 0.037) and perceived negative consequences (*p* = 0.031). Descriptively, participants with postgraduate or professional qualifications reported the highest concordance (mean 3.48 ± 0.82) and the lowest perceived negative consequences (mean 2.78 ± 0.79), whereas those with college or diploma qualifications reported the lowest concordance (mean 2.87 ± 0.79) and the highest perceived negative consequences (mean 3.36 ± 0.79). No statistically significant differences were observed for perceived benefits, practical barriers or modified BIPQ scores. No post hoc pairwise comparisons were undertaken because these analyses were exploratory and the study was not powered to detect differences between individual educational groups. Comparisons across ethnic groups showed no statistically significant differences in any of the five measures (all *p* > 0.05), although these findings should be interpreted cautiously given the small numbers in some subgroups.

## 4 Discussion

This study identifies a clear and clinical gap between high awareness of pressure injury risk and perceived benefits of preventive activities, and the actual engagement in preventive behaviours among PwSCI. Although participants demonstrated strong illness awareness and recognised the value of prevention, adherence to recommended routines, particularly pressure‐relief activities, remained only moderate. This highlights a critical limitation of knowledge‐based approaches, indicating that awareness alone is insufficient to drive sustained self‐management. Instead, engagement appears to be constrained by a combination of perceptual and practical barriers that impede the translation of intention into consistent behaviour.

These findings extend existing qualitative evidence in SCI, which has shown that cognitive beliefs, motivation and perceived feasibility are shaped by contextual challenges such as fatigue, environmental accessibility and competing demands [22, 23, 26, 27]. For example, Zanini et al. described heterogeneous ‘styles of prevention’, while Gourlan et al. highlighted the emotional dimensions such as fear, threat and acceptance that influence engagement. However, previous studies have largely been descriptive and have not quantitatively examined the mechanisms underlying this gap.

By integrating the HBM and the PPA, this study provides a novel, theory‐informed explanation of the observed behaviour gap. It demonstrates that while perceived benefit and awareness as captured by the HBM are high, these are insufficient predictors of behaviour when practical barriers and negative perceptions as captured by the PPA are present. The strong associations between perceived negatives, practical barriers and reduced concordance provide quantitative evidence that feasibility, rather than motivation alone, is a key determinant of adherence. To our knowledge, this is one of the first quantitative studies to integrate perceptual and practical factors within a single framework to explain adherence in pressure injury prevention.

Importantly, this work advances the field by moving beyond descriptive accounts to offer an empirically supported, integrated framework that explains why preventive behaviours are not consistently adopted despite high awareness. The combined framework therefore provides a more comprehensive and actionable basis for designing targeted, patient‐centred interventions to improve long‐term prevention in SCI.

### 4.1 Links to Illness Perception Theory

Our findings of positive relationship between perceived benefit and illness concern align with the Common‐Sense Model (CSM) [28], which suggests that greater perceived threat enhances belief in the efficacy of prevention. In our study, participants who viewed pressure injury as more threatening also reported stronger preventive beliefs. However, illness concern was not directly associated with concordance, in contrast to findings in the broader illness‐perception literature [25, 29, 30]. This may reflect the high practical demands of pressure injury prevention in PwSCI, where physical and environmental barriers can override motivation. Ledger et al. [31] similarly showed that preventive behaviour requires ongoing negotiation between vulnerability, self‐efficacy and contextual demands. By quantifying both attitudes and behaviours, our study extends these qualitative insights.

However, the interpretation of concordance requires consideration of the complexity of long‐term SCI self‐management. Although our questionnaire captures reported departures or adaptations from instructed pressure‐relief activities, it does not establish whether the instructions were individually appropriate, mutually agreed or feasible within a person's everyday circumstances. Adaptations or departures may reflect physical limitations, discomfort, competing demands, care arrangements, personal preferences or the need to revise an existing regimen. Lower concordance scores should therefore not be interpreted as evidence of poor motivation, unwillingness to engage in prevention or individual failure. Rather, they may identify opportunities for collaborative discussion about the suitability of current recommendations, practical barriers and unmet support needs. This interpretation is consistent with the literature emphasising the importance of understanding pressure injury prevention within the context of people's everyday lives and the interaction between individuals, carers and healthcare services [32, 33]. Future interventions should therefore support shared review and adaptation of prevention plans, rather than focus solely on improving compliance with prescribed activities.

### 4.2 Sociodemographic and Educational Influences

Participants aged ≥ 55 years reported more positive attitudes and greater engagement in preventive behaviours, whereas injury duration was not associated with adherence. This suggests that behavioural adaptation may be more closely related to life experience and established routines than to time since injury alone. However, as injury duration is closely linked to age, their independent effects cannot be fully separated in this cross‐sectional analysis. These findings contrast with qualitative evidence suggesting declining adherence over time [34], and instead support a more complex view in which self‐management behaviours are shaped by ageing, accumulated experience and changing life circumstances rather than duration alone. Clinically, this highlights the need for tailored support across the lifespan, rather than assuming a uniform trajectory of behavioural change following injury.

Prior pressure injury education was strongly associated with more positive attitudes, greater illness perception and increased engagement in pressure‐relief and skin‐inspection behaviours. This provides robust quantitative evidence that education is not only associated with improved knowledge but also with meaningful behavioural engagement. While previous studies have suggested benefits of education such as improved skin‐management skills reported by Robineau et al. [11] and the influence of emotional representations on motivation highlighted by Gourlan et al. [23] our findings extend the literature by demonstrating consistent associations across cognitive, perceptual and behavioural domains.

Individuals with previous pressure injury reported higher concordance, fewer perceived barriers, greater illness concern and more frequent skin inspection. This provides quantitative evidence that lived experience is associated with more adaptive attitudes and preventive behaviours. While prior qualitative work, such as Van Gaal et al. [35], has suggested that experience enhances awareness and vigilance, the present findings extend this by demonstrating consistent associations across behavioural, perceptual and attitudinal domains. Our results also offer empirical support for key constructs within the HBM, particularly the role of perceived severity and cues to action in driving behaviour change [18, 19], and align with the Theoretical Domains Framework (TDF), which emphasises the importance of experiential learning in shaping beliefs, environmental context and behavioural regulation [36]. Most importantly, this highlights a potential gap in prevention strategies: individuals without prior pressure injury experience may lack the experiential cues that promote engagement, highlighting the need for interventions that enhance individuals' understanding and perception of risk before adverse events occur.

In contrast to existing tools such as the Skin Management Needs Assessment Checklist (SMnac) focus primarily on knowledge and skill acquisition in pressure injury prevention, our instrument emphasises attitudinal and perceptual dimensions, including concordance, perceived benefits and barriers. While SMnac assesses capability, the current framework captures motivational and behavioural determinants of adherence, which may therefore complementary inform personalised intervention design.

Taken together, our findings suggest that awareness of risk and perceived benefit of preventive activities alone is insufficient for sustained adherence to preventive behaviours. Effective interventions must combine education, environmental support and behavioural reinforcement to translate motivation into routine practice. This study contributes to the field by quantitatively integrating self‐management attitudes, illness perceptions and preventive behaviours within a single framework, an approach that has been largely explored qualitatively in previous SCI research. By applying constructs from the HBM and PPA theoretic framework, the study provides empirical insight into how perceptual and practical factors interact to influence adherence in real‐world settings.

### 4.3 Strengths and Limitations

This study represents one of the largest quantitative investigations of pressure ulcer (pressure injury) self‐management attitudes and behaviours in people with spinal cord injury (PwSCI). By assessing both illness perceptions and preventive attitudes and behaviours using validated instruments, it identifies key gaps and sheds light on how motivation translates into action. The inclusion of participants with varied injury levels and durations supports relevance to community‐dwelling PwSCI.

Several limitations should be considered. The cross‐sectional design precludes causal inference and does not capture changes over time. Psychometric testing was exploratory and further work is needed to confirm reliability (e.g., test–retest) and responsiveness from longitudinal data. Preventive behaviours were self‐reported, introducing potential recall and social desirability bias. Recruitment through specialist centres and charities may have favoured more engaged individuals. In addition, the anonymous nature of the survey prevented comparison between respondents and non‐respondents, limiting the ability to assess potential selection bias.

The relatively high proportion of thoracic injuries in this sample likely reflects patterns seen in community‐based SCI populations, particularly among long‐term survivors. However, this may also indicate a sample skewed towards individuals with greater independence and engagement in self‐management.

Finally, pressure injury prevention is inherently multifactorial. Factors not captured in our study such as psychological status, social support, access to equipment and rehabilitation and caregiving input may also influence behaviour and risk and should be incorporated in future research.

### 4.4 Clinical Implications and Future Research

Our findings suggest that pressure injury prevention should extend beyond information provision to address the beliefs, practical circumstances and support needs that influence everyday self‐management. Education remains essential, but should be tailored to the individual and supported by opportunities to review and resolve difficulties in implementing prevention routines.

A personalised prevention model should include a structured review of current skincare and pressure‐relief practices, previous pressure injury experience, understanding of risk, perceived benefits, negative beliefs and practical barriers. The review should also consider mobility, equipment, the home environment, carer and individual priorities. Established tools assessing skin‐management knowledge and skills, such as the Skin Management Needs Assessment Checklist, could complement this attitudinal assessment.

The carers and healthcare professionals would then agree a realistic prevention plan, including appropriate pressure‐relief and skin‐inspection routines, clarification of responsibilities, strategies to overcome barriers and actions to take if skin concerns arise. This approach recognises that difficulties with prevention may reflect practical or contextual constraints rather than a lack of motivation.

Planned follow‐up by telephone, video or face‐to‐face review would provide opportunities to assess whether the plan remains workable, address changes in health, care arrangements, equipment, or the environment and revise agreed actions. A clearly identified healthcare contact and escalation pathway would enable timely advice and support. Follow‐up could also include discussion of relevant prevention technologies, such as pressure‐monitoring or digital reminder systems where appropriate. Future research should assess this model's feasibility, acceptability and potential effectiveness with PwSCI, carers and community practitioners.

Future longitudinal and intervention studies should examine changes in attitudes, illness perceptions and care circumstances; evaluate the responsiveness of the questionnaire; and assess whether personalised support improves sustained preventive behaviour and pressure injury outcomes. Incorporating objective behavioural measures alongside psychosocial and contextual data would strengthen understanding of how prevention practices change over time and inform the development of more effective, person‐centred interventions.

## 5 Conclusion

This study shows that awareness of pressure injury risk and perceived benefit of preventive activities is necessary but not sufficient for sustained preventive behaviour. Adherence to preventive activities is shaped by a dynamic interaction between beliefs, perceptions and practical constraints. Addressing this gap requires a shift from education‐focused approaches to behaviourally informed, context‐sensitive interventions that support sustained self‐management.

## Funding

This study was funded by the British Skin Foundation (012/R/22).

## Ethics Statement

Ethical approval was granted by the Health and Social Care Ethics Sub‐Committee at a national university (Ref: 25745), and by the National Research Ethics Committee/Health Research Authority (IRAS ID: 330404; REC Ref: 23/WM/0240). The study was registered on the NIHR Clinical Research Network Portfolio (CPMS ID: 60009).

## Conflicts of Interest

The authors declare no conflicts of interest.

## Supporting information


**Appendix S1:** The questionnaire used for this study.

**Appendix S2:** Pearson correlation coefficients among key attitude variables.
**Appendix S3:** Correlations between preventive behaviours and attitude domains (Pearson correlation coefficients).

## Data Availability

The data that support the findings of this study are available from the corresponding author upon reasonable request.
