# Supplementary material for: Bridging the Gap Between Awareness and Behaviour in Pressure Injury Prevention: A Cross‐Sectional Survey of Self‐Management in People With Spinal Cord Injury
Source: Int Wound J. 2026 Sep 18;23(9):e71049. doi: 10.1111/iwj.71049 (PMC13589095; doi:10.1111/iwj.71049)
Supplement: Supplementary file 1 [file IWJ-23-e71049-s001.docx]

## Appendix 2. Pearson Correlation Coefficients Among Key Attitude Variables

| **Variable** | **Concordance** | **Perceived Negative** | **Practical Barrier** | **Perceived Benefit** | **MBIPQ** |
| --- | --- | --- | --- | --- | --- |
| Concordance | — | −0.53*** | −0.56*** | +0.22** | — |
| Perceived Negative |  | — | +0.71*** | −0.27** | — |
| Practical Barrier |  |  | — | −0.18* | — |
| Perceived Benefit |  |  |  | — | +0.32*** |
| MBIPQ |  |  |  |  | — |

p < .05 (*), p < .01 (**), p < .001(***). Negative correlations indicate that higher perceived negatives or barriers are associated with lower concordance; positive correlations indicate more favorable attitudes toward prevention or greater illness awareness.

## Appendix 3. Correlations Between Preventive Behaviours and Attitude Domains (Pearson Correlation Coefficients)

| Attitude Domain | Pressure-Relief Exercise | Skin Inspection Frequency |
| --- | --- | --- |
| Concordance | +0.49*** | ns |
| Perceived Negative | −0.22** | ns |
| Practical Barrier | −0.34*** | ns |
| Perceived Benefit | +0.18* | ns |
| MBIPQ (Illness Perception) | ns | ns |

ns = not significant. p < .05 (*), p < .01 (**), p < .001 (***). Greater engagement in pressure-relief exercise was associated with more positive attitudes (higher concordance, higher perceived benefit) and fewer perceived negatives and barriers. Skin inspection frequency showed no significant correlations with any attitude domain.
