# Supplementary material for: Bridging the Gap Between Awareness and Behaviour in Pressure Injury Prevention: A Cross‐Sectional Survey of Self‐Management in People With Spinal Cord Injury
Source: Int Wound J. 2026 Sep 18;23(9):e71049. doi: 10.1111/iwj.71049 (PMC13589095; doi:10.1111/iwj.71049)
Supplement: Supplementary file 2 [file IWJ-23-e71049-s002.doc]

**Skincare self-management Questionnaire**

**ID**

Thank you for agreeing to participate in this study. We aim to look at your view and practices of skincare self-management. The questionnaire includes three parts. Part one is about your experience and practice in skincare self-management. Part 2 and part 3 explore your personal views about skincare self-management and perceptions of having a pressure ulcer. The questionnaire should take approximately 15 minutes to complete. Please answer as many questions as you can. All your responses will be treated in strict confidence.

**Before you start your questionnaire, we like to know**

Q1. When did you have a spinal cord injury?

Q2. What is the level of your injury?

Q3. How would you describe your ethnic group? (Please tick one category)

White	Asian /Asian British	Black/ Black British	Mixed

Other ethnic group

Q4. What is your gender?

Male	Female	Prefer not to tell

Q5. How old are you?

Q6. What is your current marital status?

Single, never married

Married or is living with a partner

Widowed, divorced or separated

Prefer not to answer

Q7. What is the highest level of educational qualification you have received?

Secondary school level qualifications (e.g. A level, GCSEs, O level, International Baccalaureate)

Some college/associate degree (e.g. dipnoma)

University degree (e.g. BA, BSc, Foundation degree)

Post graduate/professional degree (e.g. PCGE, masters or doctoral qualification)

Q8. Have you ever smoked?

Q9. Have you ever had any pressure ulcer since you were injured? **(Yes/No)**

If yes, please specify

__________________________________________________________________________________

**­­­­­­­­­­­­­­­­­­­­­­­Q10. Have ever had any skincare education? (Yes/No)**

If yes, please specify where and when did you have

# Part 1 Practice on skincare self-management

1.1 **Your practice (Q1 –Q9) on ‘skin inspection’**

Q1. Do you know how often you should check your skin under your sitting area? **(Yes/No)**

***If no, go to Q10. If yes, please tick one answer***

Once per day	Twice per day	More than twice per day

Twice per week	Three times per week	As often as I need

Q2**.** How do you inspect your skin? Please tick all that apply

Use a mirror	Ask a carer	See if any pink or red marks	Feel if any “hot spots’

Q3. Last week, how many days did you perform skin inspection?

I did it: 0 1 2 3 4 5 6 7 days

Q4. Last week, when you carried out your ‘skin inspection’, how did you do?

|  | **Skin inspection** | **Always** | **Sometimes** | **Never** |
| --- | --- | --- | --- | --- |
| Q5 | I used a mirror |  |  |  |
| Q6 | I asked a carer to check |  |  |  |
| Q7 | I checked skin and felt ‘hot spot’ once per day |  |  |  |
| Q8 | I checked skin and feel ‘hot spot’ twice per day |  |  |  |
| Q9 | I checked skin and feel ‘hot spot’ more than twice per day |  |  |  |

1.2 **Your practice on ‘pressure relief’ activities (Q10-Q16)**

Q10. Do you know how often to carry out ‘pressure relief’ when you sit in a wheelchair? (Yes/No)

***If yes, please tick one answer***

every 30 min	every 1 hour	e very 2 hour	as often as I need

Q11. Do you know for how long each ‘pressure relief’ manoeuvre should last at least**?** (Yes/No)

***If yes, please tick one answer***

5-10s	10-15s	15-30s	30-90s	2-3min	more than 3 min

Q12**.** How can the wheelchair ‘pressure relief’ manoeuvre be done? Please tick all that apply

Tilting or reclining	Leaning from side to side

Leaning forward	Independent ‘push ups’

Q13. Last week, how many days did you perform wheelchair ‘pressure relief’ activities?

I did it: 0 1 2 3 4 5 6 7 days

Q14. Last week, how often did you do wheelchair ‘pressure relief’ activity?

Every 30 minutes	Every 1 hour	Every two hours;

Every 3 or 4 hours; Every more than 4 hours; Whenever I remembered

Q15. Last week, when you did ‘pressure relief’, how long did it last each time?

less than 15s	15-30s	30s-1 min	1-2 min	2-3 min	more than 3 min

| 0% | 10% | 20% | 30% | 40% | 50% | 60% | 70% | 80% | 90% | 100% |
| --- | --- | --- | --- | --- | --- | --- | --- | --- | --- | --- |

Q16. What is your best guess about how much did you perform ‘pressure relief’ activities during last week compared to the recommendations?

**1.4 Concordance (Q17 –Q22) with ‘pressure relief’ activities**

Q17 – Q22. We would like to ask you about your personal views about pressure relief exercises. There are no right or wrong answers to the questions. For each statement, please tick the box which best applies to you.

|  | **Your practice of ‘pressure relief’ exercises** | **Always** | **Often** | **Sometimes** | **Rarely** | **Never** |
| --- | --- | --- | --- | --- | --- | --- |
| Q17 | I do my pressure relief exercises for a shorter time |  |  |  |  |  |
| Q18 | I do my pressure relief exercises less frequently than instructed |  |  |  |  |  |
| Q19 | I accidentally miss my pressure relief exercise |  |  |  |  |  |
| Q20 | I decide not to do my pressure relief exercises |  |  |  |  |  |
| Q21 | I do a different type of pressure relief from the exercise I have been instructed to do |  |  |  |  |  |
| Q22 | There are times when I stop doing my pressure relief exercises |  |  |  |  |  |

# Part 2. Your views about ‘pressure-relief’ regimen

|  | **Views about pressure relief exercises** | **Strongly Agree** | **Agree** | **Uncertain** | **Disagree** | **Strongly**  **Disagree** |
| --- | --- | --- | --- | --- | --- | --- |
| Q23 | Doing wheelchair ‘pressure relief’ regularly prevents pressure ulcers |  |  |  |  |  |
| Q24 | Doing wheelchair ‘pressure relief’ regularly helps me stay out of hospital |  |  |  |  |  |
| Q25 | Doing wheelchair ‘pressure relief’ regularly helps avoid prolonged periods of bed rest. |  |  |  |  |  |
| Q26 | I am taking care of myself when I do wheelchair ‘pressure relief’ regularly. |  |  |  |  |  |
| Q27 | I would prefer to do ‘pressure relief’ regularly rather than risk having a pressure ulcer. |  |  |  |  |  |
| Q28 | I feel more in control of my life if I do my ‘pressure relief’ regularly. |  |  |  |  |  |
| Q29 | Pressure relief is the best way to avoid pressure ulcers. |  |  |  |  |  |
| Q30 | Doing ‘pressure relief’ exercises are uncomfortable. |  |  |  |  |  |
| Q31 | I sometimes worry that doing ‘pressure relief’ activities regularly will cause injuries. |  |  |  |  |  |
| Q32 | Wheelchair ‘pressure relief’ get in the way of other things. |  |  |  |  |  |
| Q33 | Other lifestyle considerations such as diet and muscle exercise are more important than pressure relief activities. |  |  |  |  |  |
| Q34 | It is embarrassing to do wheelchair ‘pressure relief’ in public. |  |  |  |  |  |
| Q35 | It is a hassle to do wheelchair ‘pressure relief’ all day. |  |  |  |  |  |
| Q26 | I find it tiring to do my pressure relief exercises. |  |  |  |  |  |
| Q37 | It is difficult to keep doing ‘pressure relief’ regularly when my routine changes. |  |  |  |  |  |
| Q38 | When I’m busy it is more difficult to do pressure relief activities regularly. |  |  |  |  |  |
| Q39 | I find it difficult to remember to do my pressure relief. |  |  |  |  |  |
| Q40 | I find it difficult to find somewhere private to do pressure relief. |  |  |  |  |  |
| Q41 | I forget to do my pressure relief exercises. |  |  |  |  |  |
| Q42 | I need people around me who support me to do pressure relief activities. |  |  |  |  |  |

# Part 3 The Brief Illness Perception Questionnaire

For the following questions, please circle the number that best corresponds to your view of what it would be like if you had a pressure ulcer:

1. How much would a pressure ulcer affect your life?

0	1	2	3	4	5	6	7	8	9	10

no affect	severely

at all	affects my life

2. How long do you think a pressure ulcer would continue?

0	1	2	3	4	5	6	7	8	9	10

a very	forever

short time

3. How much control do you feel you have over whether you get a pressure ulcer?

0	1	2	3	4	5	6	7	8	9	10

absolutely	extreme amount

no control	of control

4. How much do you think treatment can help pressure ulcers?

0	1	2	3	4	5	6	7	8	9	10

not at all	extremely

helpful

5. How much do you think 'pressure relief' exercise can help prevent pressure ulcers?

0	1	2	3	4	5	6	7	8	9	10

not at all	extremely

helpful

6. How concerned are you about pressure ulcers?

0	1	2	3	4	5	6	7	8	9	10

not at all	extremely

concerned	concerned

7. How well do you feel you understand pressure ulcers?

0	1	2	3	4	5	6	7	8	9	10

don’t	understand

understand at all	very clearly

8. How much would pressure ulcers affect you emotionally? (e.g. would it make you angry, scared, upset or depressed?)

0	1	2	3	4	5	6	7	8	9	10

not at all	extremely

affected	emotionally

emotionally	affected

**Thank you for taking the time to complete this questionnaire**
